# Effects of Combined Pentadecanoic Acid and Tamoxifen Treatment on Tamoxifen Resistance in MCF−7/SC Breast Cancer Cells

**DOI:** 10.3390/ijms231911340

**Published:** 2022-09-26

**Authors:** Ngoc Bao To, Vi Nguyen-Phuong Truong, Meran Keshawa Ediriweera, Somi Kim Cho

**Affiliations:** 1 Interdisciplinary Graduate Program in Advanced Convergence Technology and Science, Jeju National University, Jeju 63243, Korea; 2 Department of Biochemistry and Molecular Biology, Faculty of Medicine, University of Colombo, Colombo 008, Sri Lanka or; 3 Subtropical−Tropical Organism Gene Bank, Jeju National University, Jeju 63243, Korea; 4 Department of Biotechnology, College of Applied Life Sciences, SARI, Jeju National University, Jeju 63243, Korea

**Keywords:** anticancer therapy, breast cancer stem cells, estrogen receptor, pentadecanoic acid, synergistic effect, tamoxifen resistance

## Abstract

Estrogen receptors are indicators of breast cancer adaptability to endocrine therapies, such as tamoxifen. Deficiency or absence of estrogen receptor α (ER−α) in breast cancer cells results in reduced efficacy of endocrine therapy. Here, we investigated the effect of combined tamoxifen and pentadecanoic acid therapy on ER−α−under−expressing breast cancer cells. Drug resistance gene expression patterns were determined by RNA sequencing analysis and in vitro experiments. For the first time, we demonstrate that the combined treatment of pentadecanoic acid, an odd−chain fatty acid, and tamoxifen synergistically suppresses the growth of human breast carcinoma MCF−7 stem cells (MCF−7/SCs), which were found to be tamoxifen−resistant and showed reduced ER−α expression compared with the parental MCF−7 cells. In addition, the combined treatment synergistically induced apoptosis and accumulation of sub−G1 cells and suppressed epithelial−to−mesenchymal transition (EMT). Exposure to this combination induces re−expression of ER−α at the transcriptional and protein levels, along with suppression of critical survival signal pathways, such as ERK1/2, MAPK, EGFR, and mTOR. Collectively, decreased ER−α expression was restored by pentadecanoic acid treatment, resulting in reversal of tamoxifen resistance. Overall, pentadecanoic acid exhibits the potential to enhance the efficacy of endocrine therapy in the treatment of ER−α−under−expressing breast cancer cells.

## 1. Introduction

Breast cancer is known to be the most commonly diagnosed cancer in women [1]. Moreover, the heterogeneity and diverse subtypes of breast cancer reduce the efficacy of breast cancer treatment [2,3]. Identifying breast cancer subtypes in patients is one of the basic steps in selecting the appropriate treatment strategy. In addition to histopathological characterization, breast cancers are classified according to the presence of estrogen receptors (ERs), progesterone receptors (PRs), and human epidermal growth factor receptor−2 positive (ERBB2/HER2+) [4]. Among the types of breast cancers, ER−α−positive breast cancer is the most frequently detected, accounting for nearly 75% of all breast cancers. ER expression has contributed to the success of hormone therapy, a mainstay of treatment for patients with ER−positive breast cancer. Indeed, loss of ER expression results in failed response to conventional hormone therapy in patients with ER−negative breast cancer [5,6,7].

Tamoxifen, an antineoplastic, nonsteroidal selective estrogen receptor modulator (SERM), is one of the most popular chemotherapeutic agents for treating ER−positive breast cancer [8]. However, development of resistance to tamoxifen has become a major clinical issue [9]. Loss of ER expression is reported to be associated with the development of tamoxifen resistance [8]. Therefore, ER−α re−expression in ER−α−negative breast cancer tumors after treatment can help overcome resistance to tamoxifen and other hormone therapies.

The main mechanism of ER signaling inactivation is the loss of ER−α gene expression [10]. Further, repression of the ER−α gene in ER−α−negative breast cancer cells was found to be caused by hypermethylation and acetylation/de−histones at the ER−α promoter [11]. Indeed, convincing evidence has demonstrated that use of epigenetic modulators, such as DNA methyltransferase (DNMT) inhibitors [12] and histone deacetylase (HDAC) inhibitors [13,14], in cancer treatment successfully re−expressed ER−α and enhanced sensitivity to endocrine therapy in ER−α−negative breast cancers. These studies indicate the crucial role of epigenetics in the regulation of ER−α gene expression.

Natural products demonstrate promising results in the treatment of various types of cancer [15]. Pentadecanoic acid (C15:0) is an odd−chain saturated fatty acid found naturally in some types of plants and fish, dairy fats, and ruminant meats [16]. A previous study conducted in our laboratory demonstrated that pentadecanoic acid reduces stemness characteristics and induces apoptosis in MCF−7/SC cells via JAK/STAT3 signaling [17]. In addition, pentadecanoic acid was found to be an HDAC6 inhibitor [18]. 

Combination treatment has been identified as a promising strategy to overcome tamoxifen resistance [19,20,21]. Here, we investigated whether combination therapy with tamoxifen and pentadecanoic acid can re−sensitize ER−α−under−expressing breast cancer cells to tamoxifen. We further examined the effect of this combination on the cell cycle, caspase−dependent apoptosis, epithelial–mesenchymal transition (EMT), and cell survival signaling pathways. Our study presents a novel approach with the potential to enhance the efficacy of endocrine therapy in the treatment of ER−α−under−expressing breast cancer cells.

## 2. Results

### 2.1. Characterization of Drug−Resistant Human Breast Cancer MCF−7/SC

In a recent study, we performed RNA sequencing and identified differentially expressed genes (DEGs) between MCF−7 and MCF−7/SC cells. Transcriptome sequencing analysis revealed a total of 4757 DEGs satisfying the conditions of fold change (FC) ≥ 2 and *p* < 0.05 between MCF−7 and MCF−7/SC cells [22]; a total of 2436 upregulated and 2321 downregulated genes were identified [22]. Among the 4757 DEGs, 41 upregulated and 34 downregulated genes were identified, overlapping with 241 genes associated with drug resistance from transcriptome sequencing (Figure S1). Further, 330 gene enrichment pathways were identified in MCF−7/SC compared to those in MCF−7 cells using KEGG analysis (Figure S2). The top 20 enriched pathways in MCF−7/SC cells are illustrated in Figure S2, wherein 5 pathways belong to metabolism and 15 pathways, including Ras, Rap1, ErbB, MAPK, and Jak/STAT, are associated with Environmental Information Processing. KEGG pathway analysis also revealed that drug−resistance−related signaling pathways, including antifolate resistance (with 10 DEGs), ABC transporters (14 DEGs), platinum drug resistance (25 DEGs), endocrine resistance (30 DEGs), and EGFR tyrosine kinase inhibitor resistance (30 DEGs), were significantly enriched in MCF−7/SC cells compared to those in the parental MCF−7 cells (Figure S3). Overall, transcriptome analysis indicated that MCF−7/SC and MCF−7 cells displayed distinct gene profiles and significant enrichment of DEGs related to drug resistance in MCF−7/SC.

**Figure 1:**
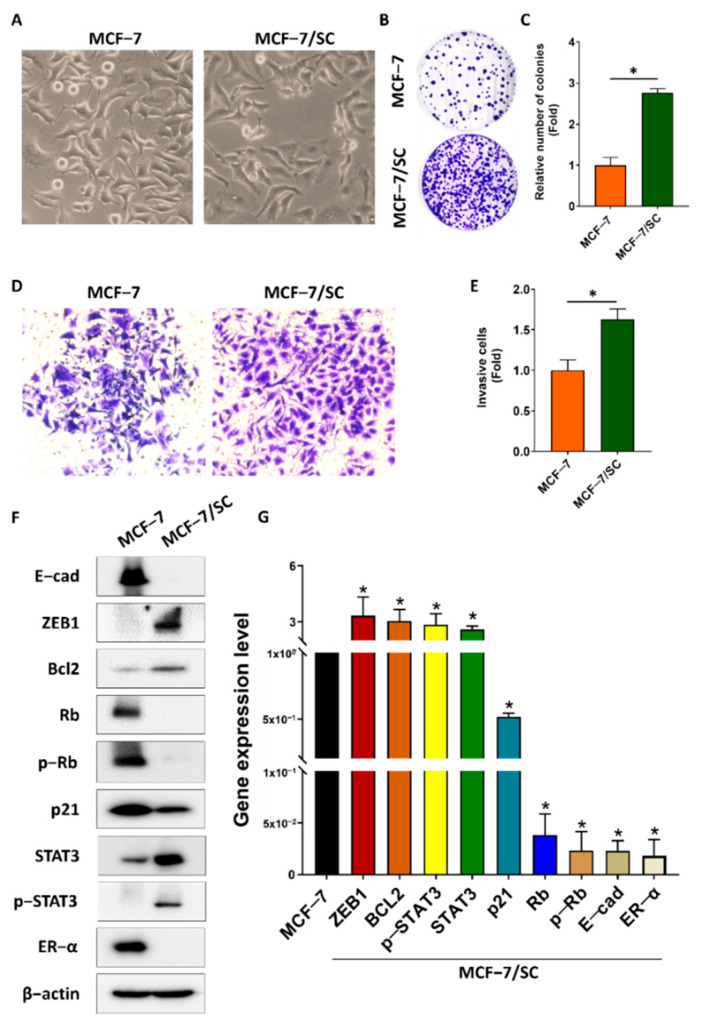
**Characterization of drug−resistant human breast cancer MCF−7/SC cells.** (**A**) Comparison of morphology between MCF−7 and MCF−7/SC cells. (**B**,**C**) The number of colonies and (**D**,**E**) invasive cells analyzed to compare MCF−7 and MCF−7/SC cells. (**F**) Representative Western blot analysis for drug resistance markers. (**G**) Real−time PCR was performed to access the gene expression. β−actin was used as a loading control. The asterisk (*) indicates *p* < 0.05 vs. the control. Data are representative of three biologically independent experiments and values are shown in mean ± SD.

The features of MCF−7/SC cells were further characterized to confirm the transcriptome analysis results. Microscopic analysis showed obvious morphological differences between the MCF−7 and MCF−7/SC cells. MCF−7 cells exhibited a round or irregular epithelial−like morphology. In contrast, MCF−7/SC cells had a more luminal, elongated shape and increased intercellular distance (Figure 1A). Clonogenic ability was evaluated using a colony formation assay. The clonogenic ability of MCF−7/SC cells was higher than that of the parental MCF−7 cells (Figure 1B,C). The results of the Transwell assay revealed the enhanced invasive capacity of MCF−7/SC cells (Figure 1D,E). To further support our hypothesis, we performed Western blotting to analyze the expression of several proteins involved in cancer drug resistance. MCF−7/SC cells expressed higher levels of Snail, Bcl−2, STAT3, and p−STAT3 and reduced expression of tumor suppressor proteins, such as Rb, p−Rb, E−cad, and p21, relative to those in the parental MCF−7 cells (Figure 1F,G). Notably, the KEGG pathway map related to the endocrine resistance pathway (hsa 01522) revealed loss of ER expression in MCF−7/SC cells (Figure S4). Consistent with this observation, we found that the expression of ER−α mRNA and protein in MCF−7/SC cells was significantly lower than that in MCF−7 cells (Figure 1F,G). These results indicate that loss of ER−α might play an important role in MCF−7/SC drug resistance. Overall, MCF−7/SC cells displayed stronger drug resistance characteristics than those of the parental MCF−7 cells.

### 2.2. Tamoxifen Resistance of MCF−7/SC Cells Compared with the Parental MCF−7 Cells

**Figure 2:**
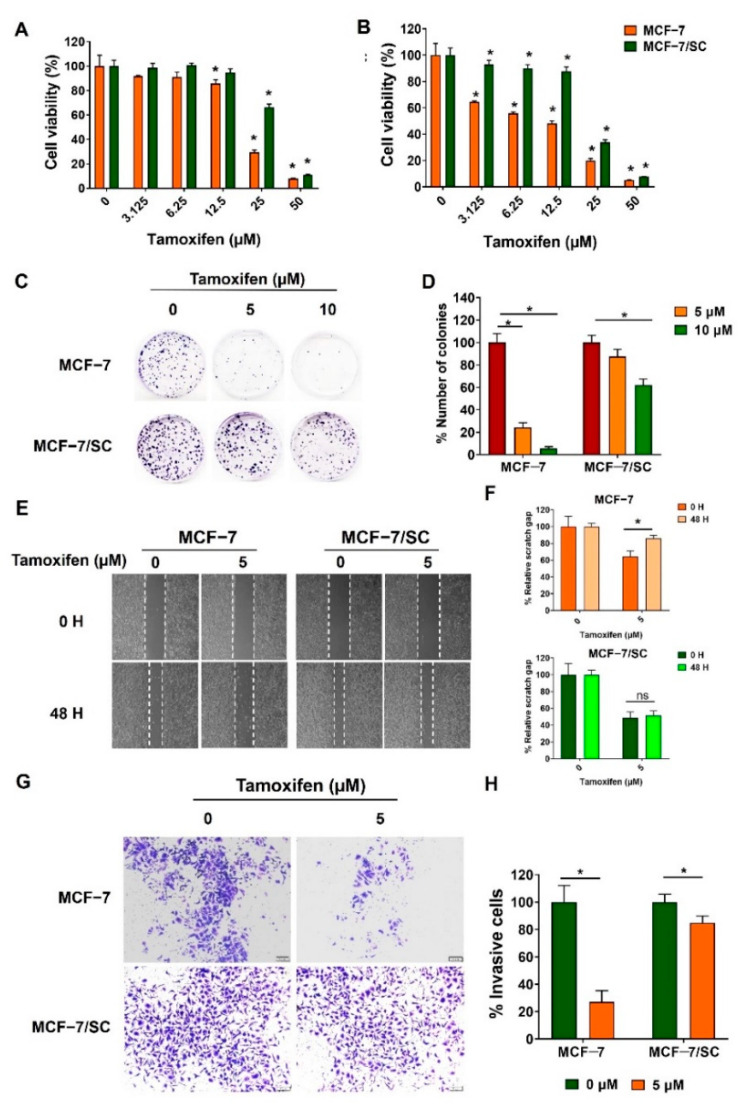

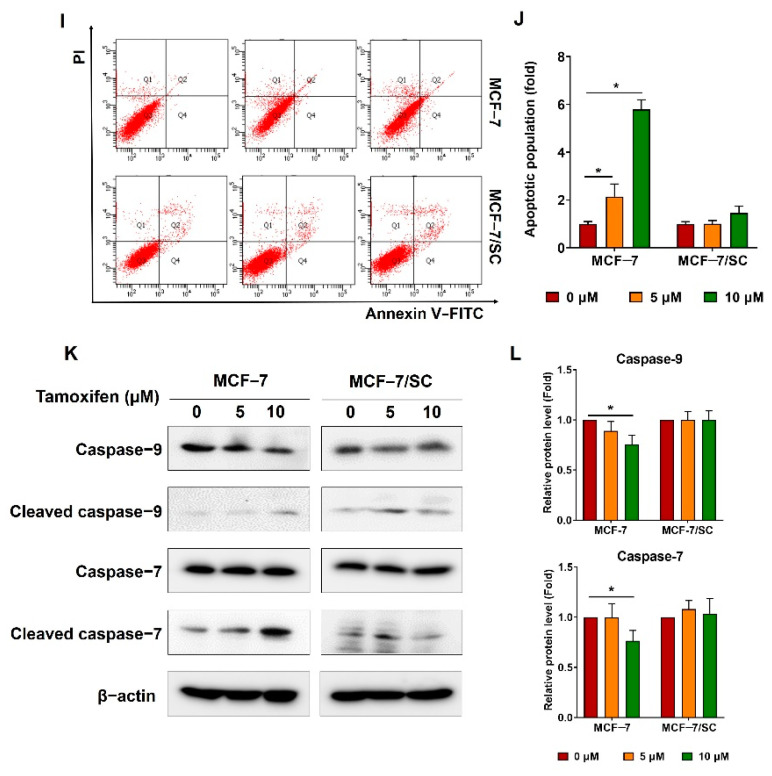
**MCF−7/SC cells displayed resistance to tamoxifen.** Viability of MCF−7 and MCF−7/SC cells was determined after tamoxifen treatment for 24 h (**A**) and 48 h (**B**). The effect of tamoxifen on (**C**,**D**) colony formation, (**E**,**F**) migration, and (**G**,**H**) invasive capacity was compared between MCF−7 and MCF−7/SC cells. (**I**,**J**) The apoptotic population detected after tamoxifen treatment. (**K**,**L**) Western blot analysis for apoptosis markers after tamoxifen exposure. β−actin was used as a loading control. The asterisk (*) indicates *p* < 0.05 vs. the control. Data were representative of three biologically independent experiments and values were shown in mean ± SD.

As the therapeutic effects of tamoxifen are primarily ER−mediated, loss of ER expression in breast cancer cells is closely related to tamoxifen resistance [23,24]. First, to examine whether MCF−7/SC cells exhibited resistance to tamoxifen, an MTT assay was performed. Tamoxifen exerted lower cytotoxicity in MCF−7/SC cells than in MCF−7 cells at 24 h and 48 h post−incubation (Figure 2A,B). Further, tamoxifen strongly inhibited the clonogenic ability of MCF−7 cells but had no effect on MCF−7/SC after 10 d of incubation (Figure 2C,D). The effect of tamoxifen was further confirmed using migration and invasion assays. As illustrated in Figure 2E–H, tamoxifen exhibited greater inhibitory effects on the migration and invasion capacities of MCF−7 cells, whereas no significant inhibitory effect was observed in MCF−7/SC. According to previous findings, tamoxifen induces apoptosis in MCF−7 cells, as confirmed by annexin V/PI staining [25]. Consistent with this finding, we confirmed that tamoxifen can induce apoptosis in MCF−7 cells; however, we did not observe this ability of tamoxifen in MCF−7/SC cells (Figure 2I,J). Indeed, apoptosis was confirmed in parental MCF−7 cells through a decrease in the full−length form and an increase in the cleaved form of the apoptosis marker proteins caspase−7 and caspase−9, which was not evident in MCF−7/SC (Figure 2K,L). Overall, these results demonstrate that MCF−7/SC cells display higher resistance to tamoxifen than do the parental MCF−7 cells.

### 2.3. Effect of Combined Pentadecanoic acid and Tamoxifen Treatment on the Growth of MCF−7/SC Cells

**Figure 3:**
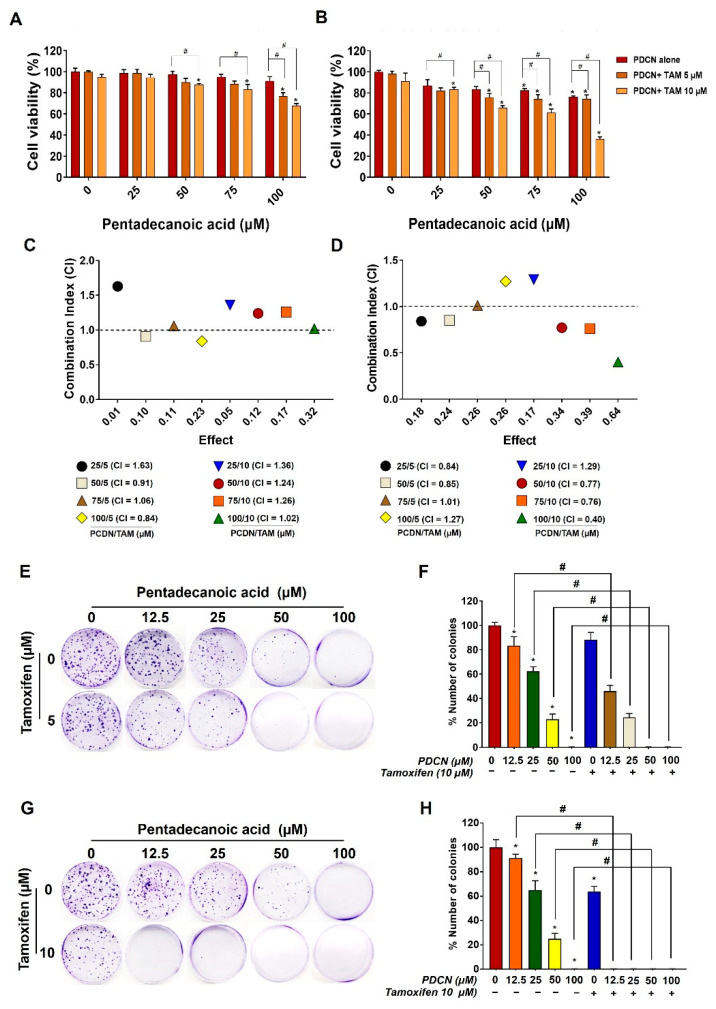
**Synergistic effect of combined treatment of pentadecanoic acid and tamoxifen in MCF−7/SC cells.** Cell viability was assessed using the MTT assay with pentadecanoic treatment alone or in combination with tamoxifen (0 μM, 5 μM, and 10 μM) at (**A**) 24 h and (**B**) 48 h. CI values were calculated after combined treatment at (**C**) 24 h and (**D**) 48 h in MCF−7/SC cells. Effects of pentadecanoic acid and tamoxifen treatment alone at 5 µM (**E**,**F**) and 10 µM (**G**,**H**) or their combination on colony formation of MCF−7/SC cells. The asterisk (*) indicates *p* < 0.05 vs. the control. The hash mark (#) indicates *p* < 0.05 when comparing combined treatment vs. individual treatment. PDCN: pentadecanoic acid; Tam: tamoxifen. The CI values illustrated in the graph are the mean from three independent experiments. Data are representative of three biologically independent experiments and values are shown in mean ± SD.

Our previous study demonstrated that pentadecanoic acid, an odd−chain fatty acid, can inhibit the proliferation of MCF−7/SC breast cancer cells [17]. Furthermore, the combination of tamoxifen with certain anticancer compounds or drugs is reported to promote antiproliferative activity in ER−α−negative breast cancer [19,20,26]. Therefore, we hypothesized that pentadecanoic acid could enhance the in vitro cytotoxic efficacy of tamoxifen in MCF−7/SC cells. Combined pentadecanoic acid and tamoxifen treatment resulted in inhibition of MCF−7/SC cell viability compared with their individual treatments (Figure 3A,B). To assess whether pentadecanoic acid and tamoxifen have synergistic cytotoxic effects in MCF−7/SC cells, combination index (CI) values were calculated. As shown in Figure 3C,D, synergism (CI < 1) was observed after combined treatment with 5 and 10 μM tamoxifen and different concentrations of pentadecanoic acid for 48 h. Combined treatment comprising 10 μM tamoxifen and 50, 75, and 100 μM pentadecanoic acid resulted in synergistic inhibitory effects with CI values of 0.77, 0.76, and 0.40, respectively (Figure 3C). These synergistic inhibitory effects were observed when pentadecanoic acid (μM) and tamoxifen (nM) were combined in the ratios of 5:1, 7.5:1, and 10:1. The combination of 10 μM tamoxifen and 25 μM pentadecanoic acid, as well as combination treatments with tamoxifen for 24 h, showed an antagonistic effect with CI values higher than 1. The colony formation assay also indicated that the combination of pentadecanoic acid (50 or 100 μM) and tamoxifen (5 or 10 μM) exhibited marked inhibitory effects on the clonogenic growth of MCF−7/SC compared with either treatment alone (Figure 3E–H). Overall, these results suggest that pentadecanoic acid effectively re−sensitized MCF−7/SC cells to tamoxifen.

### 2.4. Effect of Combined Pentadecanoic Acid and Tamoxifen Treatment on Cell Cycle in MCF−7/SC

**Figure 4:**
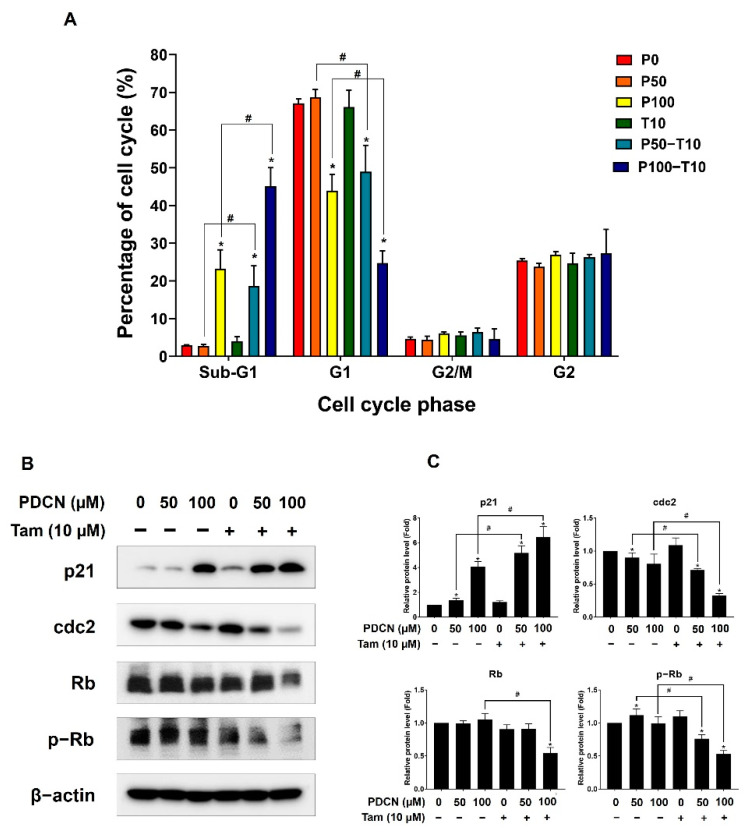
**Effect of combined treatment with pentadecanoic acid and tamoxifen on the cell cycle.** (**A**) Cell cycle analysis of MCF−7/SC exposed to pentadecanoic acid, tamoxifen, or their combination for 48 h. (**B**,**C**) The levels of cell cycle markers were assessed by Western blot experiments following treatment with pentadecanoic acid, tamoxifen, or their combination for 48 h in MCF−7/SC. β−actin was considered a loading control. The asterisk (*) indicates a *p* < 0.05 vs. control. The hash mark (#) indicates a *p* < 0.05 comparing combined treatment vs. individual treatment. P0, P50, or P100: the doses of pentadecanoic acid at 0 μM, 50 μM, or 100 μM. T10: the dose of tamoxifen at 10 μM. PDCN: pentadecanoic acid; Tam: tamoxifen. Data are representative of three biologically independent experiments and values are shown in mean ± SD.

As the combination of pentadecanoic acid and tamoxifen exerted a proliferation inhibitory effect, we assessed the effects of this combination on the cell cycle of MCF−7/SC cells. The combination of pentadecanoic acid (100 μM) and tamoxifen (10 μM) effectively arrested the cell cycle of MCF−7/SC at the sub−G1 phase (45.12 ± 4.92%) compared to the control (2.91 ± 0.19%) or that of pentadecanoic acid treatment alone (23.29 ± 5.0%) (Figure 4A). To confirm the effects of the combination of pentadecanoic acid and tamoxifen on cell−cycle−associated protein markers, we performed Western blot analysis (Figure 4B,C). The complexes of cyclin−dependent kinase (CDK), cyclins, and retinoblastoma (Rb) are well−known regulators of the cell cycle [27]. The combination of pentadecanoic acid and tamoxifen significantly suppressed the level of cdc2, a CDK for G2/M transition, and p−Rb, a CDK substrate, whereas it drastically increased the level of p21, a CDK inhibitor in MCF−7/SC, compared to those in the control or pentadecanoic acid treatment alone (Figure 4B,C). Collectively, these data demonstrate that pentadecanoic acid combined with tamoxifen could arrest the cell cycle by accumulating the sub−G1 population.

### 2.5. Effect of Combined Pentadecanoic Acid and Tamoxifen Treatment on Apoptosis in MCF−7/SC

**Figure 5:**
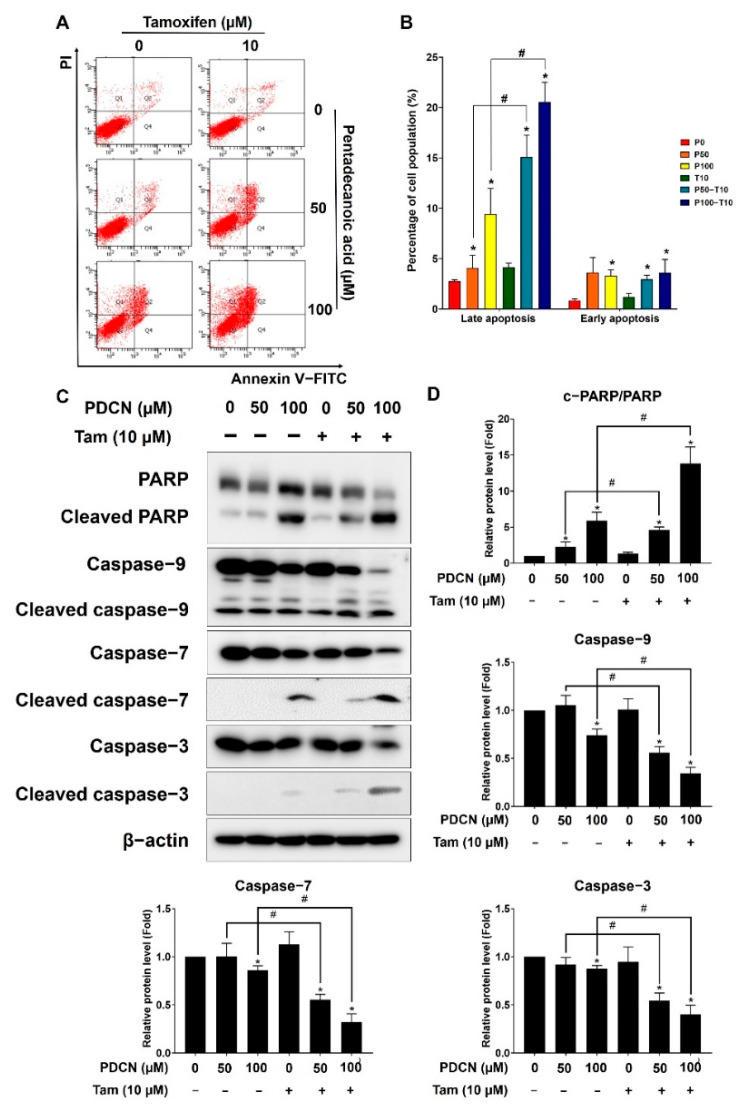
**Pentadecanoic acid combined with tamoxifen induces apoptosis.** (**A**,**B**) AnnexinV/PI staining was used to determine the apoptotic population following treatment with pentadecanoic acid or tamoxifen alone in combination for 48 h. (**C**,**D**) The levels of apoptosis markers were assessed by Western blot analyses following individual or combined treatment for 48 h in MCF−7/SC cells. β−actin was used as a loading control. The asterisk (*) indicates a *p* < 0.05 vs. the control. The hash mark (#) indicates a *p* < 0.05 when comparing the combined vs. individual treatments. P0, P50, or P100: the doses of pentadecanoic acid at 0 μM, 50 μM, or 100 μM. T10: the dose of tamoxifen at 10 μM. PDCN: pentadecanoic acid; Tam: tamoxifen. Data are representative of three biologically independent experiments and values are shown in mean ± SD.

As an increase in the sub−G1 cell population is associated with the promotion of apoptosis [28], we examined whether the combination of pentadecanoic acid and tamoxifen could induce apoptosis in MCF−7/SC cells. The results of annexin V/PI staining indicated no significant effect of 10 µM tamoxifen on apoptosis, whereas pentadecanoic acid treatment alone triggered cell death via late apoptosis in MCF−7/SC cells (Figure 5A,B). Notably, the combination of pentadecanoic acid (100 μM) and tamoxifen (10 μM) promoted a seven−fold increase in the late apoptotic population compared with that in the untreated group or with tamoxifen alone, which was nearly two−fold higher than that obtained with pentadecanoic acid treatment alone in MCF−7/SC cells. This combination also significantly increased the percentage of early apoptotic cells compared to that in the control but not that in the pentadecanoic acid alone treatment. To further elucidate the effect of the combination of pentadecanoic acid and tamoxifen on apoptosis, Western blot analysis was performed. Consistent with the annexin V/PI staining results, the combination of pentadecanoic acid and tamoxifen treatment increased the cleavage of PARP, caspase 3, caspase 7, and caspase 9 and reduced the uncleaved form of these apoptosis−associated proteins (Figure 5C,D). These results suggest that the combination of pentadecanoic acid and tamoxifen induces apoptosis in MCF−7/SC cells. 

### 2.6. Effect of Combined Pentadecanoic Acid and Tamoxifen Treatment on Epithelial–Mesenchymal Transition

**Figure 6:**
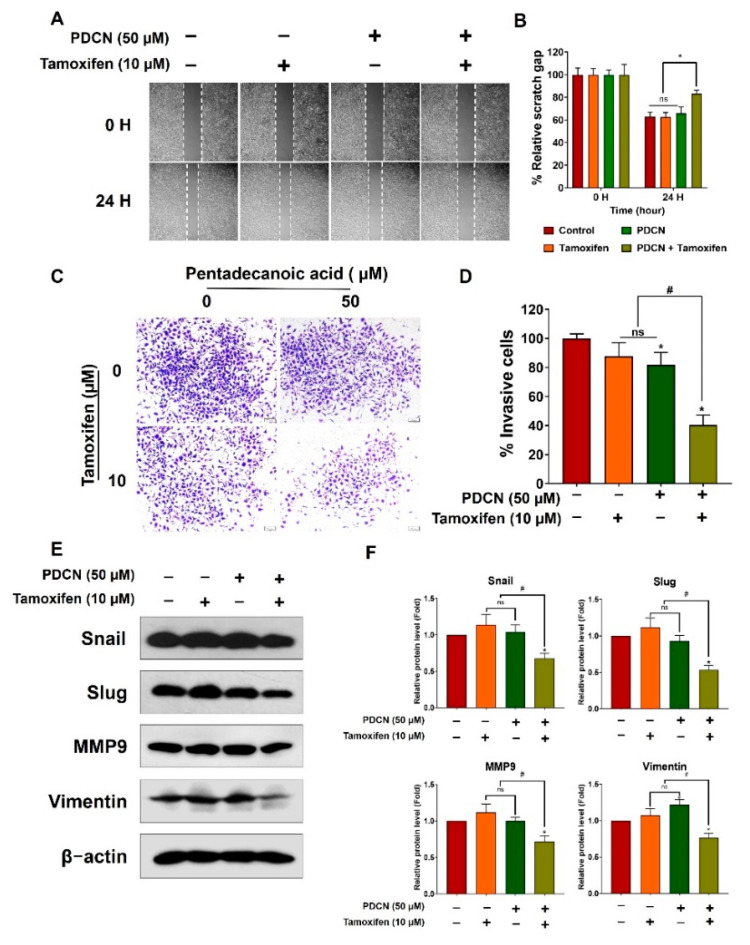
**Combined treatment with pentadecanoic acid and tamoxifen suppressed EMT.** (**A**,**B**) Cell migration determined using the wound healing assay following treatment for 48 h in MCF−7/SC cells. (**C**,**D**) Invasive cells determined after pentadecanoic acid treatment alone or in combination with tamoxifen for 48 h. (**E**,**F**) Western blot analysis of epithelial–mesenchymal transition (EMT) markers in MCF−7/SC were performed after individual or combined treatment for 48 h. β−actin was used as a loading control. The asterisk (*) indicates *p* < 0.05 vs. the control. The hash mark (#) indicates *p* < 0.05 when comparing the combined vs. individual treatments. PDCN: pentadecanoic acid. Data are representative of three biologically independent experiments and values are shown in mean ± SD.

Previous studies have demonstrated the crucial role of epithelial–mesenchymal transition (EMT) in cancer cell metastasis and drug resistance [29,30]. Therefore, we evaluated the influence of the pentadecanoic acid and tamoxifen combination on EMT using migration assays, invasion assays, and Western blot analysis. Combined treatment with 50 µM pentadecanoic acid and 10 µM tamoxifen significantly inhibited the migration and invasion capacities of MCF−7/SC cells (Figure 6A–D). These observations were further confirmed by Western blot analysis for EMT−associated markers, such as Snail, Slug, matrix metalloproteinase 9 (MMP9), and vimentin. Individual treatment with tamoxifen or pentadecanoic acid did not show a significant inhibitory effect on the expression of these proteins (Figure 6E–F). In contrast, the combined treatment drastically decreased the expression of EMT−related proteins in MCF−7/SC cells. Overall, these data demonstrate that the combination of pentadecanoic acid and tamoxifen can effectively suppress migration and invasion capacity and decrease the expression of EMT−associated markers in MCF−7/SC cells.

### 2.7. Effect of Combined Pentadecanoic Acid and Tamoxifen Treatment on ER−α Expression in MCF−7/SC Cells

**Figure 7:**
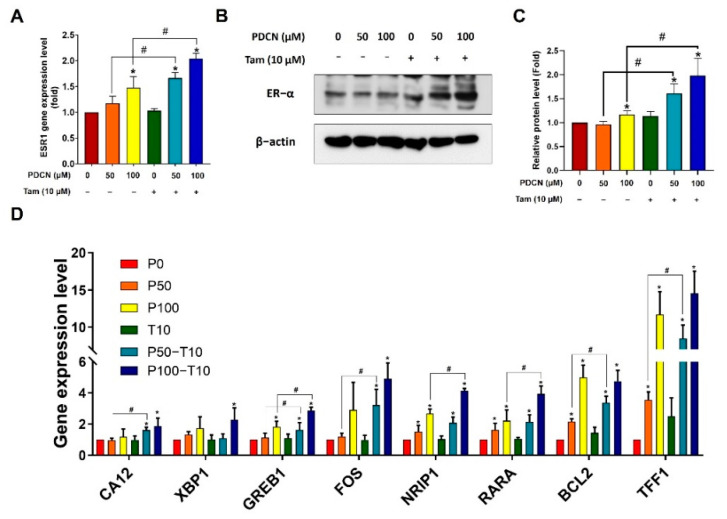
**Pentadecanoic acid induces ER−α expression in MCF/7/SC cells**. (**A**) Gene expression of ER−α after pentadecanoic acid treatment alone or in combination with 10 μM tamoxifen. (**B**,**C**) The protein level of ER−α was determined after pentadecanoic acid treatment alone or in combination with 10 μM tamoxifen by Western blot analysis in MCF/7/SC cells. (**D**) The expression of ER−α−related genes after 48−h treatment with pentadecanoic acid alone or in combination with 10 μM tamoxifen in MCF−7/SC cells. β−actin was used as a loading control. PDCN: pentadecanoic acid; Tam: tamoxifen. P0, P50, or P100: the doses of pentadecanoic acid at 0 μM, 50 μM, or 100 μM. T10: the dose of tamoxifen at 10 μM. The asterisk (*) indicates *p* < 0.05 vs. the control. The hash mark (#) indicates *p* < 0.05 when comparing the combined vs. individual treatments. Data are representative of three biologically independent experiments and values are shown in mean ± SD.

Lack of ER expression has been reported to be involved in tamoxifen resistance [31]. Therefore, we hypothesized that the combination of pentadecanoic acid and tamoxifen may induce ER−α expression in ER−α−under−expressing breast cancer MCF−7/SC cells. To examine this, we first treated MCF−7/SC cells with pentadecanoic acid (50 or 100 μM), alone or in combination with 10 μM tamoxifen, and examined ER−α expression. As expected, pentadecanoic acid alone or in combination with tamoxifen restored ER−α expression at both the mRNA and protein levels (Figure 7A–B). We further investigated whether this combination affected other ER−α−related genes. Pentadecanoic acid alone or in combination could induce the expression of ER−α−related genes, including CA12, XBP1, GREB1, FOS, NRIP1, RARA, BCL2, and TFF1 (Figure 7C). These data indicate that re−expression of ER−α by pentadecanoic acid alone or in combination contributes to restoration of tamoxifen sensitivity and reversal of tamoxifen resistance in ER−under−expressing MCF−7/SC breast cancer cells.

ER−α suppression has been associated with epigenetic modifications, particularly involving class I and II HDACs [26,32]. Notably, we previously demonstrated that pentadecanoic acid is a novel HDAC6 inhibitor [18]. Therefore, we investigated whether the restoration of ER−α expression by the combined treatment with pentadecanoic acid and tamoxifen was modulated through epigenetic events in MCF−7/SC. Western blot analysis revealed that pentadecanoic acid treatment alone or in combination with tamoxifen can dramatically enhance the acetylation of intracellular α−tubulin, a well−known HDAC6 substrate, in MCF−7/SC cells (Figure S5A,B). In conclusion, these results indicate that pentadecanoic acid alone or in combination with tamoxifen may reactivate ER−α expression by modulating α−tubulin acetylation.

### 2.8. Effect of Combined Pentadecanoic Acid and Tamoxifen Treatment on Cell Survival Signaling Pathways

**Figure 8:**
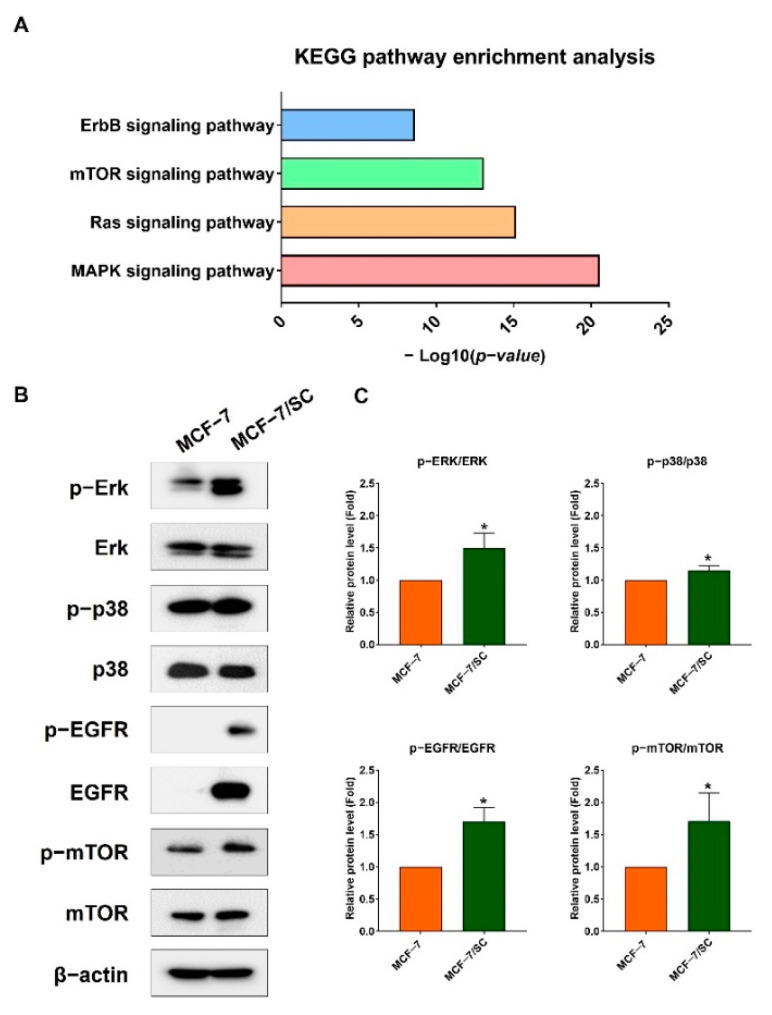

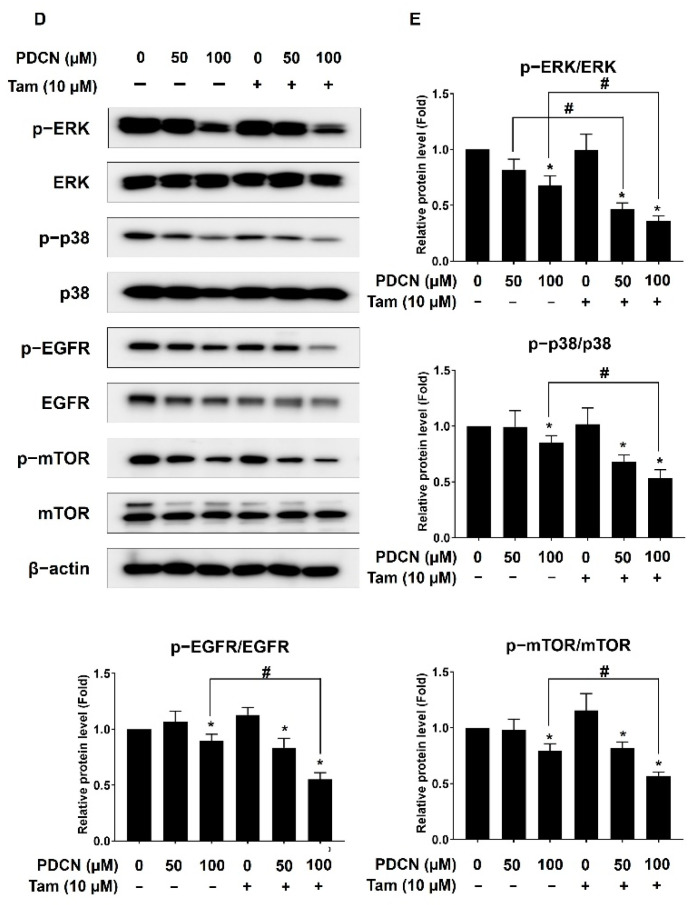
**Pentadecanoic acid combined with tamoxifen inhibited survival−associated signaling pathways.** (**A**) KEGG analysis was used to analyze the enrichment of common survival pathways in MCF−7/SC cells compared to those in MCF−7 cells. The levels of proteins related to ERK1/2, MAPK, EGFR, and mTOR signaling compared between MCF−7 and MCF−7/SC (**B**,**C**) cells or following pentadecanoic acid or combined treatment for 48 h in MCF−7/SC cells (**D**,**E**). β−actin was used as a loading control. The asterisk (*) indicates *p* < 0.05 vs. the control. The hash mark (#) indicates *p* < 0.05 when comparing the combined vs. individual treatments. Data are representative of three biologically independent experiments and values are shown in mean ± SD.

Various components of the EGFR, mTOR, Ras, and MAPK signaling pathways have become attractive targets for anticancer therapy [33,34]. Further, the public data from the Kaplan–Meier plotter indicate that high gene expression of EGFR, mTOR, MAPK, and ERK1/2 is positively associated with shorter relapse−free survival (RFS) in ER−negative breast cancer patients (Figure S6A–D). Consistent with previous reports and Kaplan–Meier plotter analysis, KEGG analysis indicated an enrichment of survival signaling pathways in MCF−7/SC compared with those in MCF−7 cells (Figure 8A). Notably, the Ras, MAPK, and ErbB signaling pathways were among the top 20 signaling pathways enriched in MCF−7/SC cells (Figure S2). We confirmed these observations by comparing the protein levels of ERK1/2, p38, mTOR, and EGFR in MCF−7 and MCF−7/SC cells. As expected, the levels of p− ERK1/2, p−p38, p−mTOR, and p−EGFR in MCF−7SC cells were higher than those in MCF−7 cells (Figure 8B–C). The protein levels of ERK1/2, p38, and mTOR were similar between the two cell lines, whereas the EGFR levels were remarkably lower in MCF−7 cells than in MCF−7/SC cells (Figure 8B,C). As the combination of pentadecanoic and tamoxifen inhibited cell proliferation, we examined whether this combination could suppress the levels of ERK1/2, MAPK, EGFR, and mTOR signaling pathway components in MCF−7/SC cells; this combination decreased the ratio of the phosphorylated form to the total form of ERK1/2, MAPK, EGFR, and mTOR protein levels (Figure 8D,E). Collectively, these results demonstrate that the combination of pentadecanoic acid and tamoxifen can suppress critical survival signaling pathways, such as ERK1/2, MAPK, EGFR, and mTOR signaling in MCF−7/SC cells. 

## 3. Discussion

Heterogeneity is known to be a characteristic feature of breast cancer [35]. Therefore, the response of breast cancer to each treatment is disparate and depends mainly on the alteration of molecular expression, such as the estrogen receptor status [36,37,38]. Indeed, ER overexpression—a marker required for endocrine therapy response—has been observed in approximately two thirds of the patients with breast cancer [39]. Meanwhile, patients with ER−negative breast cancer exhibit a malignant phenotype and have poor prognosis; further, endocrine therapy does not yield viable outcomes in these patients [40]. Based on these observations, re−sensitization to endocrine therapy is a potential strategy for treating ER−negative breast cancer. In this study, reduced ER−α expression and significantly high tamoxifen resistance were observed in MCF−7/SC cells compared to in the parental MCF−7 cells. In this respect, MCF−7/SC could be considered a suitable cell line for studies related to regulation of ER expression to overcome tamoxifen resistance in breast cancer.

However, single−agent chemotherapeutic compounds have significant limitations in that they are not individually as effective, or are effective only at dose levels that are unsafe for patients [21]. Considering these challenges, combination therapy is a favorable approach for cancer treatment. Combinations of cancer therapies can be used to create a synergistic effect using more than one drug when each drug has a different target signaling pathway. Further, combination therapy increases the treatment success rate by reducing the concentration of individual drugs. Thus, the combination of two or more compounds is an effective approach to increase the success rate and reduce the concentration of individual drugs in therapeutic regimens [41]. In this study, we investigated the combination of pentadecanoic acid and tamoxifen at nonlethal concentrations to examine whether pentadecanoic acid could overcome tamoxifen resistance in tamoxifen−resistant MCF−7/SC cells. Pentadecanoic acid combined with tamoxifen inhibited the proliferation of MCF−7/SC cells, with CI values ranging from 0.40 to 0.77. Consistent with their proliferation inhibitory effect, the colony−forming capacity of MCF−7/SC cells was significantly inhibited after exposure to this combination. Furthermore, the results indicated that combined treatment with pentadecanoic acid and tamoxifen resulted in cell cycle arrest at the sub−G1 phase. Consistent with previous studies showing that modulating the cell cycle could induce or inhibit apoptosis [42], the combination of pentadecanoic acid and tamoxifen induced apoptosis through caspase−dependent activation, indicating that pentadecanoic acid synergizes with tamoxifen treatment. 

Tamoxifen resistance has been of constant interest to scientists; however, the molecular mechanism of tamoxifen resistance remains unclear. In addition to pharmacological systems, overexpression of coactivators or suppression of corepressors that regulate ER transcription, absence or change in ER expression, and activation of various signaling pathways involved in cell survival and proliferation, EMT is considered a contributing factor to tamoxifen resistance [43,44]. In particular, loss of ER expression has been reported to cause EMT [45]. Among EMT−inducing transcription factors, Slug, ZEB1, and Snail are positively correlated with tamoxifen resistance [46,47]. Snail can suppress ER−α expression via direct interaction with a regulatory DNA sequence at the ESR1 locus, which encodes ER−α protein [48]. Another member of the Snail family, Slug, has been shown to protect tamoxifen−resistant breast cancer cells via the slug/hexokinase 2 signaling pathway [49]. In a study conducted on patients with breast cancer, a correlation was observed between ER−α and two typical EMT markers, MMP9 and vimentin [50]. Consistent with the reported results, our data showed that pentadecanoic acid overcomes tamoxifen resistance by inhibiting EMT and suppressing migratory and invasive behavior, as well as protein expression of EMT markers, such as Snail, Slug, ZEB1, and vimentin in MCF−7/SC cells.

A negative correlation between EGFR and ER−α has been reported in many studies, and EGFR is frequently overexpressed in ER−negative breast cancer cell lines [51,52,53,54]. Previous studies have shown that dimerization of EGFR and HER2 leads to the rapid activation of downstream kinases, such as Akt, PCK−α, and ERK1/2−MAPK, leading to the loss of ER−α expression in breast cancer cells [55,56]. EGFR overexpression, also known as an indicator of resistance to hormone therapy, is associated with decreased response to tamoxifen [57,58]. However, to explore the underlying mechanisms by which tamoxifen can increase the expression of ER−α target genes and pentadecanoic acid can act as an ER−α agonist, experiments with ER−α siRNA and a selective estrogen receptor degrader (fulvestrant) should be conducted. Further, analysis of clinical data showed a positive correlation between HDAC and mTOR in patients with triple−negative breast cancer [20]. Another study revealed that mTOR inhibition restored the response to tamoxifen in resistant cell lines [59]. Therefore, inhibition of signaling pathways, such as EGFR, ERK1/2, and mTOR, is required to overcome resistance to endocrine therapies, including resistance to tamoxifen. Consistent with these findings, our transcriptome analysis revealed EGFR overexpression and activation of the MAPK, mTOR, and PI3K−Akt signaling pathways in MCF−7/SC cells. Furthermore, the protein levels of EGFR, p−EGFR, p−mTOR, and p−ERK1/2 were significantly higher than those in tamoxifen−sensitive MCF−7 cells. However, combined treatment with pentadecanoic acid and tamoxifen markedly suppressed ERK1/2, MAPK, EGFR, and mTOR signaling by downregulating the expression of ERK1/2, MAPK, EGFR, and mTOR, and reducing the phosphorylated form of these proteins in MCF−7/SC cells.

Further, epigenetic events are known to contribute to ER silencing and enhance resistance to endocrine therapies, such as tamoxifen [32,40,60,61]. Indeed, class I and class II HDACs are involved in the suppression of ER−α as transcriptional repressors through histone modification events in ER−negative breast cancer, which do not benefit from hormone−receptor−based treatment [26,32,62]. Therefore, use of HDAC inhibitors is a promising approach to elevate ER−α expression contributing to the re−sensitization of ER−negative breast cancer cells to endocrine therapies. Pentadecanoic acid was found to act as an HDAC6 inhibitor in our previous study [18]. Notably, pentadecanoic acid treatment alone or in combination with tamoxifen elevated the expression of *ESR1* gene, ER−related genes, and ER−α protein in MCF−7/SC cells. Together with ER−α re−expression, our results showed a drastic increase in the protein expression of acetylated α−tubulin, a well−known substrate of HDAC6. A previous report demonstrated that hyperacetylation of α−tubulin using HDAC6 inhibitors can affect microtubule stability, leading to cellular sensitization to anticancer drugs through activation of the caspase system and promotion of apoptosis [63]. We found that apoptotic population and cleaved form of apoptosis−associated caspase cascade markers, such as caspase 3, caspase 7, caspase 9, and PARP, were significantly increased in MCF−7/SC upon combined treatment with pentadecanoic acid and tamoxifen. Based on the important role of HDACs in both the gene expression and enzyme activity involved in the regulation of ER transcriptional activity, many studies have successfully used HDAC inhibitors to reverse the ER status in ER−negative breast cancer cells. Among the HDACs identified to be involved in ER transcription and regulation of ER expression, HDAC6 is a novel and promising candidate for the re−sensitization of ER−negative cell lines to hormone therapy [64]. Recently, the association between membrane−localized ER/HDAC6/α−tubulin has been demonstrated in ER−positive breast cancer cell lines [65]. However, no study has investigated this association in ER−negative breast cancer cell lines. Our study is the first to indicate that α−tubulin modification may contribute to ER−α restoration by the combined pentadecanoic acid and tamoxifen treatment, particularly in ER−α−under−expressing breast cancer cells. However, further studies are needed to elucidate how pentadecanoic acid and tamoxifen combination is involved in the restoration of ER−α through epigenetic modulation. An ideal animal model should be established to evaluate the effectiveness of pentadecanoic acid, as well as its combination with tamoxifen in innovational breast cancer treatments. Pentadecanoic acid and tamoxifen dosages have been shown to be safe for normal cells in an in vitro model (data not shown); however, it is imperative to re−evaluate this safety using animal models.

## 4. Materials and Methods

### 4.1. Cell Lines and Cell Culture

Human estrogen−receptor−positive breast cancer cells (MCF−7) and breast cancer stem−like cells (MCF−7/SC) were used in this study. MCF−7 cells were cultured according to instructions from the American Type Culture Collection (ATCC, Rockville, MD). MCF−7 cells were cultured in DMEM supplemented with 10% FBS, 100 U/mL of penicillin, and 100g/mL of streptomycin according to instructions from the American Type Culture Collection (ATCC, Rockville, MD). MCF−7/SC cells were generated from parental MCF−7 cells by sorting CD44+/CD24−/dim cell population [66] and characterized in our previous studies [17,22]. RPMI 1640 supplemented with 10% FBS, 100 U/mL of penicillin, and 100 g/mL of streptomycin was used for growing the MCF−7/SC cells. The proliferation of MCF−7 and MCF−7/SC is ~29 h and 25 h, respectively.

### 4.2. Cell Viability Assay

Cells (4000 cells/well) were seeded in 96−well cell culture plates with the indicated culture media and incubated at 37 °C for the MTT assay. Following incubation, cells were treated for 48 h with various dosages of pentadecanoic acid, tamoxifen, or both. After 24 and 48 h of incubation, the MTT assay was performed and cell viability was determined as described previously [17,22]. 

### 4.3. Colony Formation Assay

MCF−7 and MCF−7/SC cells (400 cells/dish) were cultured for 24 h. Following culture, the cells were exposed to pentadecanoic acid, tamoxifen, or both. After incubation for 10 d, the colonies were fixed with 4% paraformaldehyde and stained with 2% crystal violet. Stained colonies were manually counted and expressed as percentages compared to untreated controls for each tested concentration. 

### 4.4. Wound Healing Assay

MCF−7 and MCF−7/SC cells (1 × 10^5^ cells/dish) were cultured in 6−well plates until they formed monolayers. A uniform scratch was made through the confluent cell monolayers using a sterile pipette tip, and the cell layer was washed with PBS. The cells were then exposed to pentadecanoic acid, tamoxifen, or a combination of both for 24 h and 48 h. After incubation, the widths of the scratches were measured using a phase−contrast microscope (×100). 

### 4.5. Cell Invasion Assay

As described previously [15,20], a 24−well Transwell system (0.2 m pore; Corning, Inc., New York, NY, USA) was used for the cell invasion assay. Pentadecanoic acid and tamoxifen, alone or in combination, were added to the upper chambers in FBS−free media. Media with 10% FBS was added to the lower chambers. After 48 h of incubation, the cells were fixed with 4% paraformaldehyde and stained with 2% crystal violet. Invading cells were then observed under a phase−contrast microscope (100×).

### 4.6. Flow Cytometry

Flow cytometry was used to investigate the effects of the treatments on the cell cycle and apoptosis, as previously described [15,20]. Cells (1 × 10^5^ cells/dish) were exposed to pentadecanoic acid and tamoxifen, alone or in combination, for 48 h. BD FACSDiva™ Software (BD Biosciences, Franklin Lakes, NJ, USA) was used to analyze the apoptosis population. An Annexin V−FITC Apoptosis Detection Kit (BD Biosciences) was used to detect apoptosis. Cell cycle analysis was carried out by ACScalibur flow cytometry (Becton Dickinson, Franklin Lakes, NJ, USA in Bio-Health Materials Core-Facility, Jeju National University) and the percentage of each population in cell cycle was analyzed and graphed by using GraphPad Prism 7 (GraphPad Software, Inc., La Jolla, CA, USA).

### 4.7. Western Blotting

Radioimmunoprecipitation assay (RIPA) buffer was used to prepare cell lysates from MCF−7 or MCF−7/S cells (3 × 10^5^ cells/dish) after drug treatment, and BCA assay (Thermo Fisher Scientific, Waltham, MA, USA) was used to determine protein quantity. Following sample preparation, proteins were separated based on molecular weight (MW) using sodium dodecyl sulfate−polyacrylamide gel electrophoresis (SDS−PAGE). The procedures for membrane transfer, membrane blocking, primary antibody incubation, secondary antibody incubation, and protein detection were performed as outlined in previous studies [15,20]. Antibody dilution was performed according to the manufacturer’s instructions. All primary antibodies used for Western blot are listed in Table S1.

### 4.8. Quantitative Reverse Transcription PCR (qRT−PCR)

MCF−7/SC (10^6^ cells/dish) were cultured overnight, followed by treatment with pentadecanoic acid and tamoxifen for 48 h, either separately or in combination. TRIzol^®^ Reagent was used to extract total RNA after drug exposure (Invitrogen; Thermo Fisher Scientific, Inc., Carlsbad, CA, USA). Total RNA was then used for complementary DNA (c−DNA) synthesis. Real−time PCR was then set up in a 20 µL reaction volume comprising 1 µL of c−DNA, 2 µL of the indicated primers (1 µL of each primer), 10 µL of master mix (Takara, Shiga, Japan), and 7 µL of RNA−free water. Real−time PCR was then performed under the following cycling conditions: A 15−min initial hold at 95 °C, 40 cycles of 95 °C for 10 s and 60 °C for 30 s, followed by generation of a dissociation curve at 95 °C for 15 s, 60 °C for 30 s, and a gradual increase in temperature up to 95 °C for 15 s. Primer sequences of the target genes are listed in Table S2. Gene expression was quantified using the 2^−ΔΔCq^ method [67].

### 4.9. Transcriptomic Analysis

Transcriptome analysis of MCF−7 and MCF−7/SC cells was performed in a recent study conducted in our laboratory [22]. Total RNA was extracted from MCF−7 and MCF−7/SC cells using a TRIzol kit (Invitrogen, Carlsbad, CA, USA). An Illumina TruSeq mRNA Sample Prep kit (Illumina, San Diego, CA, USA) was used to create the RNA library. To prepare for sequencing, mRNA was purified using magnetic beads with Poly−T oligo attachments. RNA−Seq was performed by Macrogen, Inc. (Seoul, Korea), in accordance with the manufacturer’s instructions. A genomic DNA reference (UCSC hg19) was used to apply the cDNA fragment obtained through the RNA sequencing. The HISAT2 program was used to align the read mapping through the Bowtie 2 aligner. Signaling pathways were investigated using the Kyoto Encyclopedia of Genes and Genomes (KEGG) Automatic Annotation Server (KAAS).

### 4.10. Correlation Analysis Using the Kaplan–Meier Plotter

The public database from the Kaplan–Meier plotter data analysis was used to examine the correlation between the gene expression levels of EGFR, mTOR, MAPK, and ERK1/2 and the prognosis of patients with breast cancer [7].

### 4.11. Combination Index Analysis

The effects of pentadecanoic and tamoxifen in drug interactions have been calculated and are represented by a combination index (CI). The following formula was used to calculate the combination index analysis, which was based on the median−effect principle; combination index(CI) = D1/(Dx)1 + D2 /(Dx)2; the denominators included (Dx)1 and (Dx)2, which represent the concentration of the first drug (Drug1) and the second drug (Drug2) alone that inhibits x%, respectively. In the numerators, D1 and D2 represented for the portion the first drug (Drug1) and the second drug (Drug2) in combination (D1 + D2) that have the same effect with the concentration of that drug in the denominators (also inhibits x%). CompuSyn software (ComboSyn, Inc.) was used to calculate the CI values according to the Chou–Talalay model [68]. Indicators of additive impact, synergism, and antagonism are CI = 1, 1, and >1, respectively.

### 4.12. Statistical Analysis

Statistical analysis was performed using GraphPad Prism 7 (GraphPad Software, Inc., La Jolla, CA, USA). The mean and standard deviation (SD) of three independent experiments were used to represent the results. For group comparisons, one−way analysis of variance (one−way ANOVA) with Dunnett’s post hoc test was used, and *p* < 0.05 was considered statistically significant.

## 5. Conclusions

Collectively, the combination of pentadecanoic acid and tamoxifen showed synergistic activity in breast cancer stem cells. This combination promoted apoptosis and resulted in cell cycle arrest at the sub−G1 phase. Moreover, pentadecanoic acid plays a prominent role in the re−expression of ER−α. Finally, suppression of EMT and inhibition of tamoxifen−resistance−associated signaling pathways were observed upon combined treatment with pentadecanoic acid and tamoxifen, indicating the potential synergistic efficacy of this approach. Overall, our observations provide the first evidence that pentadecanoic acid combined with tamoxifen can reverse the ER−α−negative status in breast cancer and enhance the efficacy of endocrine therapy. Modulating the acetylation of the HDAC6 substrate tubulin may involve epigenetic processes, leading to ER reactivation and TAM re−sensitization. The outcomes of the in vivo model will serve as the cornerstone for designing clinical trials to evaluate the efficacy of this combination in breast cancer patients.

## Data Availability

All data are available within the article and in the Smentary Materials.

## Supplementary Materials

The following supporting information can be downloaded at: www.mdpi.com/article/10.3390/ijms231911340/s1.
