# Supplementary material for: Effects of Combined Pentadecanoic Acid and Tamoxifen Treatment on Tamoxifen Resistance in MCF−7/SC Breast Cancer Cells
Source: Int J Mol Sci. 2022 Sep 26;23(19):11340. doi: 10.3390/ijms231911340 (PMC9570034; doi:10.3390/ijms231911340)
Supplement: Supplementary file 1 [file ijms-23-11340-s001.zip › ijms-1868658-supplementary.pdf]

Effects of Combined Pentadecanoic acid and Tamoxifen Treatment on Tamoxifen Resistance in MCF−7/SC Breast Cancer Cells

Supplementary Material

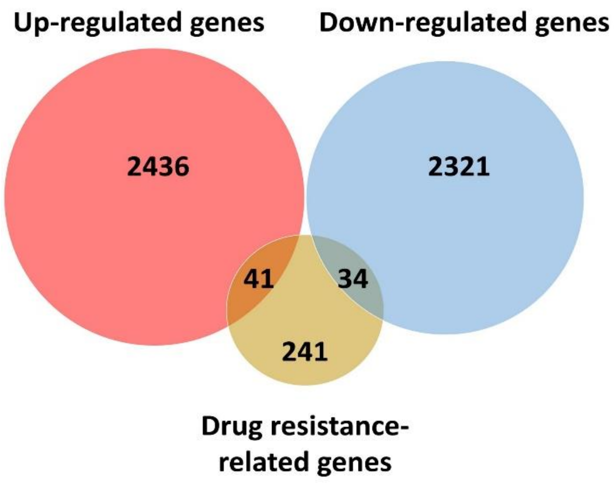

Figure S1. Venn diagram showing the number of overlapping genes between the upregulated and downregulated genes for drug−resistant markers.

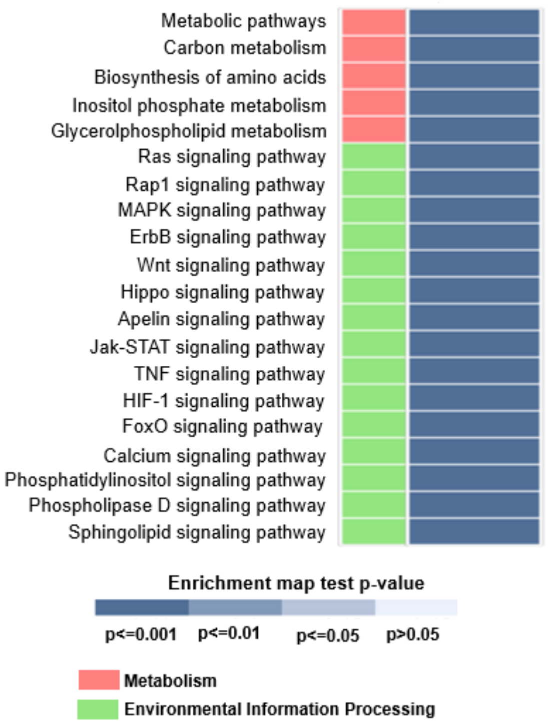

Figure S2. The top 20 pathways enriched in MCF−7/SC cells compared with those in MCF−7 cells.

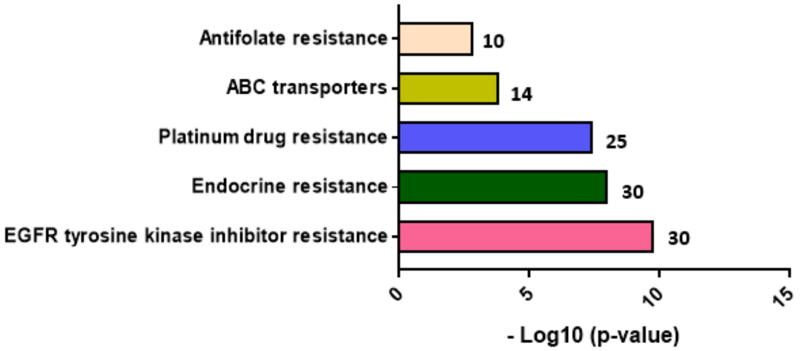

Figure S3. KEGG pathway analysis indicating the number of DEGs belonging to each of the enrichment–resistant pathways in MCF–7/SC compared with those in MCF–7 cells.

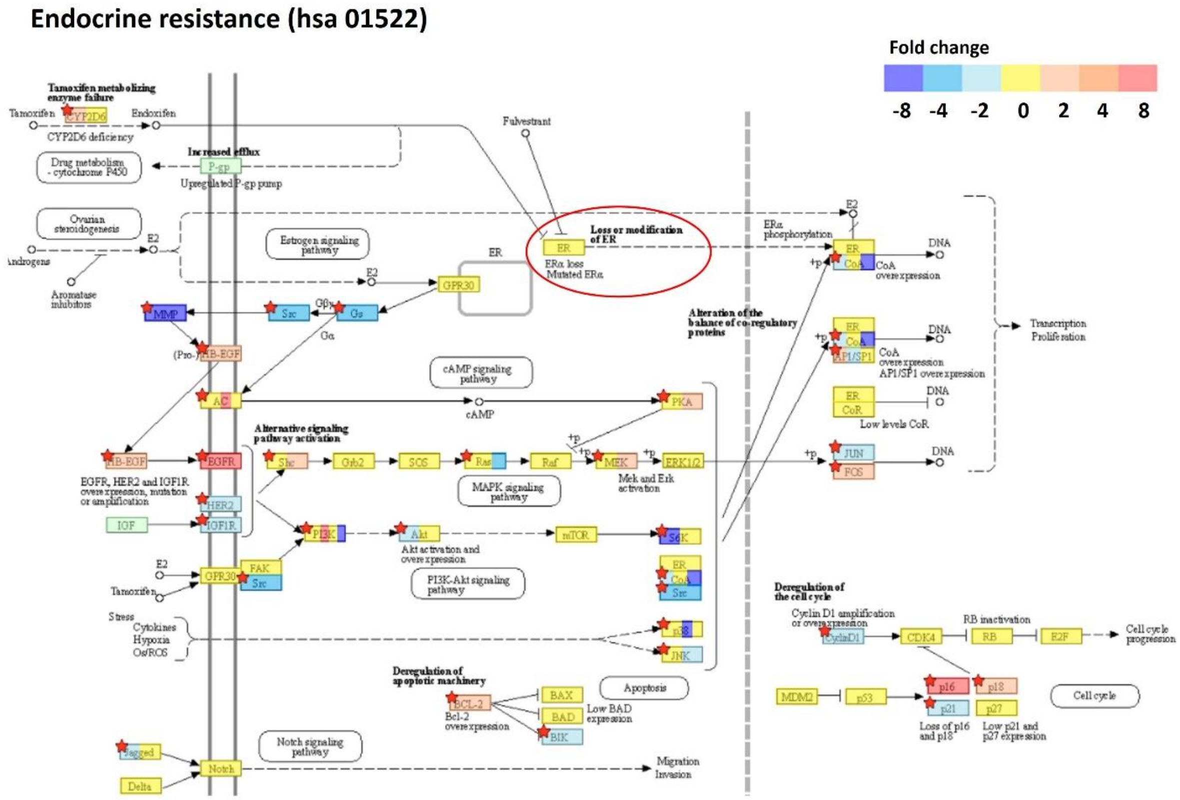

Figure S4. KEGG pathway map: Endocrine signaling pathway

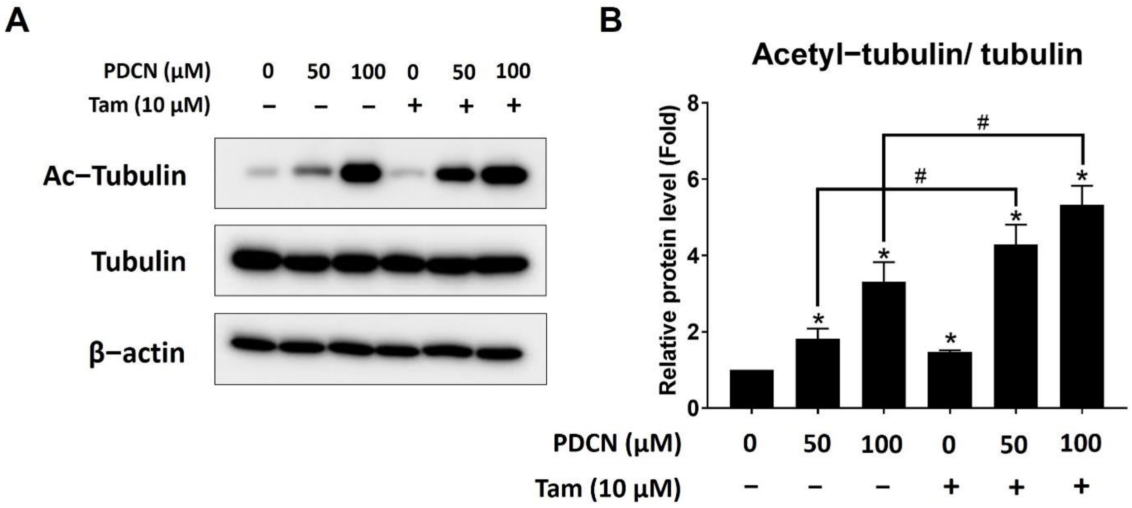

Figure S5. Pentadecanoic acid combined with tamoxifen enhanced the acetylation of intracellular α−tubulin.

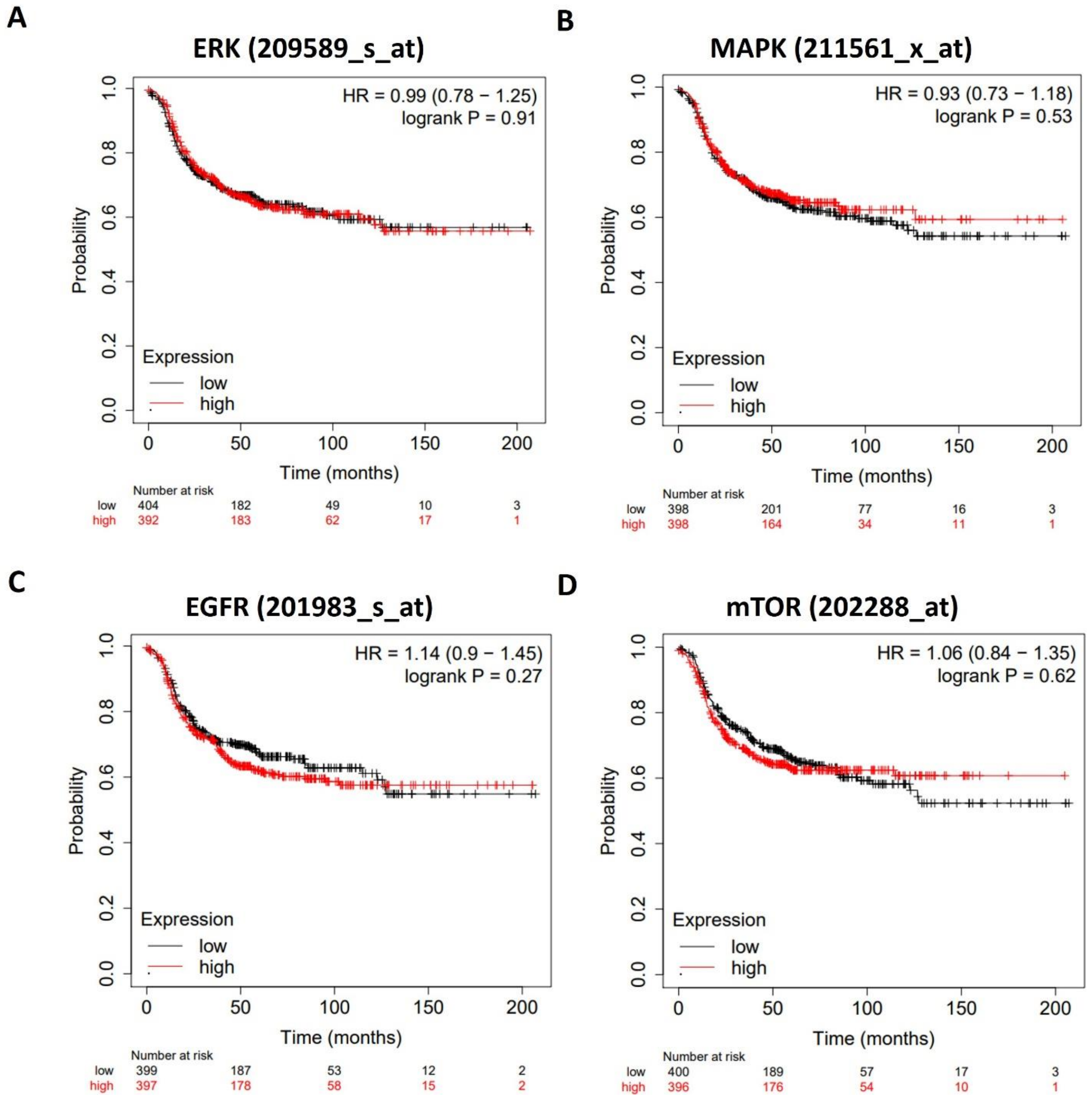

Figure S6. Analysis of ERK, MAPK, EGFR, mTOR expression in ER−negative breast cancer patients.

Table S1. The antibody list for Western blot

Antibody	*Dilution ratio*	*Catalog number and Company*
B−actin	1:10000	#3700 (Cell Signaling Technology)
E−cadherin	1:1000	610181 (BD Biosciences)
ZEB1	1:500	#3396 (Cell Signaling Technology)
Bcl2	1:1000	#4223 (Cell Signaling Technology)
Rb	1:1000	#9309 (Cell Signaling Technology)
p−Rb	1:1000	#9308 (Cell Signaling Technology)
p21	1:1000	#2947 (Cell Signaling Technology)
cdc2	1:1000	#9116 (Cell Signaling Technology)
ER−α	1:500	#8644 (Cell Signaling Technology)
Caspase 9	1:1000	#9502 (Cell Signaling Technology)
Cleaved− Caspase 9	1:1000	#9505 (Cell Signaling Technology)
Caspase 7	1:1000	#9492 (Cell Signaling Technology)
Cleaved− Caspase 7	1:1000	#9491 (Cell Signaling Technology)
Caspase 3	1:1000	#9662 (Cell Signaling Technology)
Cleaved− Caspase 3	1:500	#9661 (Cell Signaling Technology)
PARP	1:1000	#9542 (Cell Signaling Technology)
Snail	1:1000	#3879 (Cell Signaling Technology)
Slug	1:500	#9585 (Cell Signaling Technology)
MMP9	1:1000	#3852 (Cell Signaling Technology)
Vimentin	1:1000	#5741 (Cell Signaling Technology)
STAT3	1:1000	#9139 (Cell Signaling Technology)
p−STAT3	1:500	#9145 (Cell Signaling Technology)
Erk1/2	1:1000	#9102 (Cell Signaling Technology)
p− Erk1/2	1:1000	#9101 (Cell Signaling Technology)
p38	1:500	#9212 (Cell Signaling Technology)
p−p38	1:500	#9211 (Cell Signaling Technology)
EGFR	1:1000	#2232 (Cell Signaling Technology)
p−EGFR	1:500	#2234 (Cell Signaling Technology)
mTOR	1:1000	#2983 (Cell Signaling Technology)

p−mTOR	1:500	#2971 (Cell Signaling Technology)
Tubulin	1:1000	#2144 (Cell Signaling Technology)
Ac−Tubulin	1:1000	#5335 (Cell Signaling Technology)

Table S2. The primer sequences for qRT−PCR

Genes	***Primer sequences***	
ESR1	Forward (5’−>3’)	AGCTCCTCCTCATCCTCTCC
	Reverse (5’−>3')	TCTCCAGCAGCAGGTCATAG
CA12	Forward (5’−>3’)	TGGCATTCTTGGCATCTGTA
	Reverse (5’−>3')	TTGGTGGCTGGCTTGTAAAT
NRIP1	Forward (5’−>3’)	TCGCACTCACCACAGAAAAC
	Reverse (5’−>3')	AGCCAAGCTCTTCTCCATGT
RARA	Forward (5’−>3’)	CGCTGCTGGAGGCGCTAAAG
	Reverse (5’−>3')	GCGCTGATGCTTCGCAGGTC
XBP1	Forward (5’−>3’)	GCGCCTCACGCACCTG
	Reverse (5’−>3')	GCTGCTACTCTGTTTTTCAGTTTCC
TFF1	Forward (5’−>3’)	GTGTCACGCCCTCCCAGT
	Reverse (5’−>3')	GGACCCCACGAACGGTG
GREB1	Forward (5’−>3’)	CAAAGAATAACCTGTTGGCCCTGC
	Reverse (5’−>3')	GACATGCCTGCGCTCTCATACTTA
FOS	Forward (5’−>3’)	CGTCTTCCTTCGTCTTCACC
	Reverse (5’−>3')	GTCAGAGGAAGGCTCATTGC
BCL2	Forward (5’−>3’)	TCCCTCGCTGCACAAATACTC
	Reverse (5’−>3')	ACGACCCGATGGCCATAGA
